# Prolyl Carboxypeptidase Activity Is Present in Human Adipose Tissue and Is Elevated in Serum of Obese Men with Type 2 Diabetes

**DOI:** 10.3390/ijms232113529

**Published:** 2022-11-04

**Authors:** Emilie De Hert, Kenneth Verboven, Kristiaan Wouters, Johan W. E. Jocken, Ingrid De Meester

**Affiliations:** 1 Laboratory of Medical Biochemistry, Faculty of Pharmaceutical, Biomedical and Veterinary Sciences, University of Antwerp, 2610 Wilrijk, Belgium; 2 REVAL—Rehabilitation Research Center, Faculty of Rehabilitation Sciences, Hasselt University, 3590 Diepenbeek, Belgium; 3 BIOMED—Biomedical Research Center, Faculty of Medicine and Life Sciences, Hasselt University, 3590 Diepenbeek, Belgium; 4 Cardiovascular Research Institute Maastricht (CARIM), Department of Internal Medicine, Maastricht University Medical Centre+, 6229 ER Maastricht, The Netherlands; 5 Department of Human Biology, NUTRIM School of Nutrition and Translational Research in Metabolism, Maastricht University Medical Centre+, 6229 ER Maastricht, The Netherlands

**Keywords:** diabetes, human adipose tissue, metabolic disorders, obesity, prolyl carboxypeptidase

## Abstract

Prolyl carboxypeptidase (PRCP) is involved in metabolic disorders by hydrolyzing anorexigenic peptides. A link between serum PRCP activity and obesity has been reported, but its origin/source is still unclear. Previously proven correlations between human serum PRCP activity and the amount of adipose tissue may suggest that adipose tissue is an important source of circulating PRCP. We investigated PRCP activity in visceral, subcutaneous adipose tissue (VAT and SCAT), skeletal muscle tissue and serum of lean and obese men with or without type 2 diabetes (T2D). Correlations between PRCP activity, metabolic and biochemical parameters and immune cell populations were assessed. PRCP activity was the highest in VAT, compared to SCAT, and was very low in skeletal muscle tissue in the overall group. Serum PRCP activity was significantly higher in T2-diabetic obese men, compared to lean and obese non-diabetic men, and was positively correlated with glycemic control. A positive correlation was observed between serum PRCP activity and VAT immune cell populations, which might indicate that circulating PRCP activity is deriving rather from the immune fraction than from adipocytes. In conclusion, PRCP activity was observed in human adipose tissue for the first time and serum PRCP activity is correlated with T2D in obese men.

## 1. Introduction

Prolyl carboxypeptidase (PRCP, EC 3.4.16.2), a lysosomal carboxypeptidase, is of interest in metabolic disorders due to its role in the C-terminal cleavage of α-melanocyte stimulating hormone (MSH) 1–13 and (pyr)-apelin-13 [1,2,3]. α-MSH 1–13 is an anorexigenic peptide, derived from the polypeptide precursor proopiomelanocortin (POMC), that suppresses appetite and stimulates metabolism by acting on postsynaptic melanocortin receptors MC3R and MC4R in the hypothalamus [4]. PRCP cleaves off the C-terminal amino acid from α-MSH 1–13 to form α-MSH 1–12, which is inactive [1]. (Pyr)-apelin-13 originates from the precursor preproapelin, and is one of the endogenous ligands of the G-protein-coupled receptor APJ [5]. Apelin is an adipokine, a bioactive mediator secreted by adipocytes, thereby considering apelin-APJ signaling pathways as being promising therapeutic targets in metabolic diseases, like type 2 diabetes (T2D) and obesity [6,7,8]. For example, the apelin peptide increases glucose uptake and insulin sensitivity [7]. PRCP cleaves (pyr)-apelin-13 to form (pyr)-apelin-13_(1–12)_ [2,3]. Although many efforts were made in the past to elucidate the exact role of the C-terminal Phe of (pyr)-apelin-13, it is still not clear whether (pyr)-apelin-13 is a more active isoform than (pyr)-apelin-13_(1–12)_ [3,9]. PRCP gene trap mice have higher hypothalamic α-MSH 1–13 levels and a leaner phenotype compared to controls. Furthermore, they consume significantly less food and are resistant to diet-induced obesity [1,10,11]. PRCP-inhibitors show body weight reduction in diet-induced obese mice [12,13]. Interestingly, male and female mice showed differences in glucose metabolism upon PRCP-knockdown [10]. In humans, different studies were conducted investigating PRCP protein and activity levels in obesity, diabetes and cardiovascular dysfunction [14,15,16]. Plasma PRCP protein levels were increased in patients with T2D and/or obesity and were significantly correlated with signs of obesity and diabetes and markers of lipid metabolism [14]. Moreover, PRCP protein levels were significantly elevated in plasma of five uncontrolled T2D patients, while T2D patients on anti-diabetic drugs had almost normal PRCP levels [16]. These findings were supported by PRCP activity measurements in serum of female subjects with different Body Mass Index (BMI) [15]. Serum PRCP activity of the group with elevated BMI was significantly higher compared to the PRCP activity in the lean group and was correlated with plasma PRCP protein concentrations and metabolic parameters, such as adipose tissue mass. Weight loss in obese individuals, either by diet or bariatric surgery, caused a significant decrease in mean serum PRCP activity and positive correlations were found between the decrease in serum PRCP activity and the decrease in body weight, BMI, visceral, subcutaneous and total abdominal adipose tissue [15]. The correlations with the amount of adipose tissue may suggest that adipose tissue is an important source of circulating PRCP and that PRCP is secreted from adipocytes; however, to date, no data on PRCP activity in human adipose tissue are available.

To the best of our knowledge, PRCP activity has never been measured in human adipose tissue before. Therefore, we aimed to measure PRCP activity in paired subcutaneous and visceral adipose tissue (SCAT and VAT, respectively) as well as skeletal muscle tissue (m. rectus abdominis) of obese men with and without T2D as compared to lean men. Furthermore, we investigated PRCP activity in serum from these individuals. Potential correlations between PRCP activity and different biochemical and metabolic parameters were determined. To study the correlation between PRCP activity in serum and adipose tissue, we investigated in vitro whether adipocytes are capable of secreting PRCP by measuring PRCP activity in conditioned media of differentiated human multipotent adipose-derived stem cells (hMADS). Finally, since obese adipose tissue is often characterized with increased inflammation, we assessed whether PRCP activity is correlated with immune cell populations in the stromal vascular fraction (SVF) of SCAT and VAT in order to gain more insight into the potential source(s) of circulating PRCP. PRCP is already described in several diseases with an inflammatory component such as rheumatoid arthritis, stroke and sepsis [17,18,19]. Furthermore, an upregulation of PRCP mRNA expression was seen in human lipopolysaccharide-stimulated endothelial cells [20]. Pro-inflammatory stimulation of human endothelial cells also induced PRCP secretion, while its intracellular activity level remained constant [3]. Moreover, PRCP activity is already described in different immune cells like macrophages, T-cells and B-cells [15]. As chronic inflammation is described in metabolic diseases like obesity and T2D, it is of interest to study the correlation between PRCP and inflammatory or immune components in these patients.

In this study, we demonstrate for the first time that PRCP activity is present in human adipose tissue. An association was observed between serum PRCP activity and glycemic control in obese men. In both lean and obese individuals, PRCP activity was significantly higher in SCAT and VAT as compared to the activity in muscle tissue. Moreover, a positive correlation was observed between serum PRCP activity and VAT immune cell populations, which might indicate that circulating PRCP activity is deriving rather from the immune fraction than from adipocytes.

## 2. Results

### 2.1. PRCP Activity Is Higher in Adipose Tissue Compared to Skeletal Muscle Tissue

**Figure 1:**
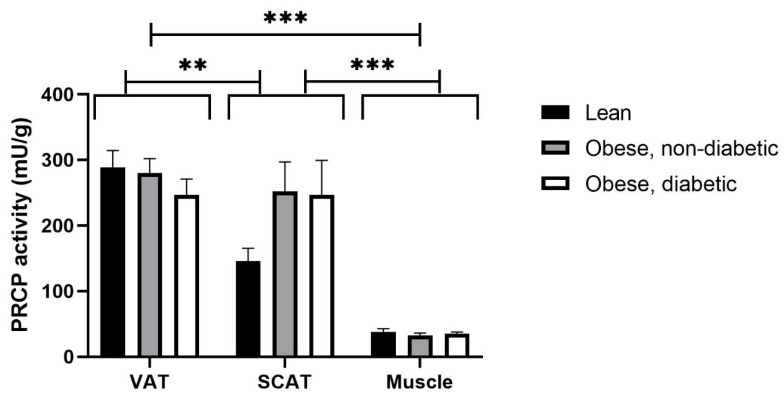
PRCP activity in VAT, SCAT and muscle tissue of lean subjects (black bars, *n* = 18 for VAT, *n* = 14 for SCAT and *n* = 16 for muscle tissue) and obese subjects without T2D (grey bars, *n* = 24 for VAT, *n* = 16 for SCAT and *n* = 12 for muscle tissue) and obese subjects with T2D (white bars, *n* = 11 for VAT, *n* = 7 for SCAT and *n* = 5 for muscle tissue). Results are presented as mean ± SEM. Significant differences are indicated on the graphs (** *p* < 0.01, *** *p* < 0.001).

As shown in Figure 1, PRCP activity is the highest in VAT, as compared to the activity in SCAT, and is only minimally present in muscle tissue. Two-way ANOVA analysis showed no significant interaction between the effects of tissue type (VAT, SCAT and skeletal muscle tissue) and group (obese, with and without T2D, and lean individuals) (*p* = 0.106). A main effect was seen for tissue type (*p* < 0.001), but not for group (*p* = 0.477). Post hoc analysis showed a significant difference in PRCP activity between VAT and SCAT (*p* = 0.007), between VAT and muscle tissue (*p* < 0.001) and between SCAT and muscle tissue (*p* < 0.001) for the overall group. Although the data on Figure 1 suggest that the PRCP activity in SCAT seems to be higher in obese subjects compared to lean subjects, no main effect was reported for the group, indicating that no significant differences were seen between the lean group, the obese group with T2D and the obese group without T2D (*p* = 0.477).

**Figure 2:**
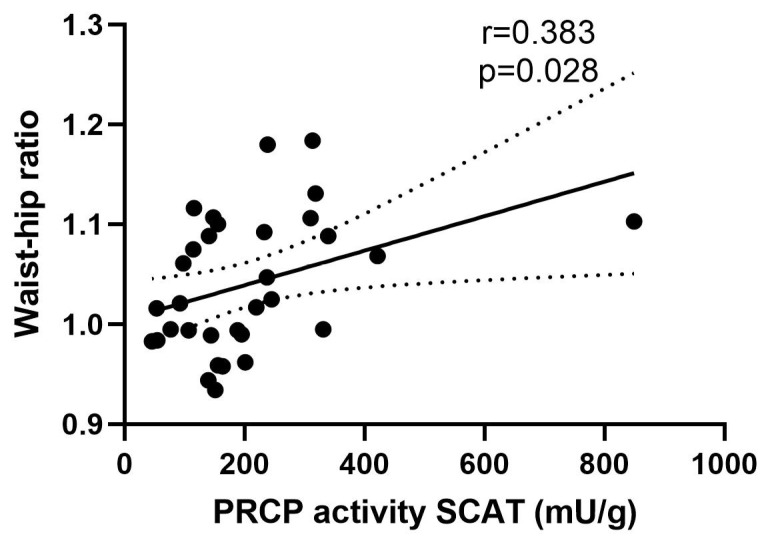
Correlation between PRCP activity in SCAT and waist–hip ratio. Linear regression line is shown with 95% confidence interval (r = 0.383 and *p* = 0.028). Circles: individual values, solid line: Linear regression line; dotted line: 95% confidence interval for the regression line.

A significant correlation (r = 0.383; *p* = 0.028, Figure 2) between PRCP activity in SCAT and the waist-hip ratio was observed in the overall group. No other significant correlations between PRCP activity in SCAT and other metabolic or biochemical parameters could be found. No significant correlations between PRCP activity in VAT or muscle tissue and metabolic or biochemical parameters could be observed either.

**Figure 3:**
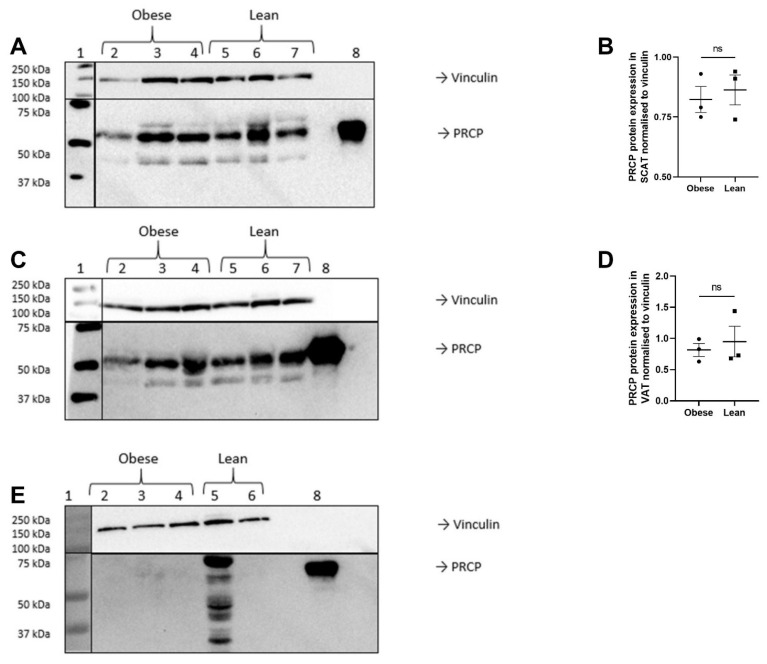
PRCP expression in SCAT (**A**,**B**), VAT (**C**,**D**) and muscle tissue (**E**) of lean and obese individuals. Lane 1: Precision Plus Protein Dual Color Standards; Lane 2–3: tissue of 2 different obese individuals without T2D; Lane 4: tissue of an obese individual with T2D; Lane 5–7: tissue of 3 different lean individuals (except for Figure 2C: Lane 7 is empty); Lane 8: rhPRCP as positive control. Vinculin was visualized for the use as loading control. The Western blot on muscle tissue showed nonspecific binding of the PRCP antibody in one sample. This sample showed visually detectable contamination with blood, contrary to the other muscle samples. The lines indicate different parts of the membrane that were incubated with a different antibody or detected with another system (white light for the Standards and chemiluminescence for the antibodies). Quantification of PRCP protein expression was performed in relation to vinculin. No significant differences in PRCP protein expression were observed between lean and obese individuals in SCAT or VAT (ns = not significant). Circles: PRCP protein expression for obese individuals; Squares: PRCP protein expression for lean individuals.

PRCP protein expression in SCAT (Figure 3A,B) and VAT (Figure 3C,D) of obese and lean subjects was confirmed via Western blot. Quantification of PRCP protein expression was performed in relation to vinculin. No significant differences in PRCP protein expression were observed between lean (*n* = 3) and obese individuals (*n* = 3) in SCAT or VAT. The low number of samples available for Western blotting obliged us to combine obese and obese, diabetic individuals, which limits the added value of the statistical comparison between groups. In contrast, PRCP protein could not be detected in muscle tissue via Western blot (Figure 3E).

### 2.2. Serum PRCP Activity Is Elevated in Obese Men with T2D

**Figure 4:**
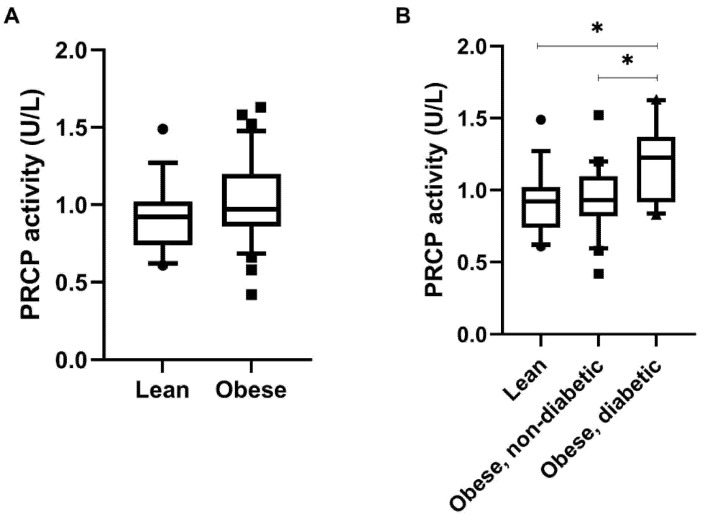
Serum PRCP activity (U/L) in lean and obese men with or without T2D. (**A**) PRCP activity measured in serum of lean men (*n* = 19) and obese men (*n* = 31). (**B**) PRCP activity measured in serum of lean men (*n* = 19), obese men without T2D (*n* = 21) and obese men with T2D (*n* = 10) (* *p* < 0.05). Results are presented as boxplots with 10–90 percentile. Significant differences are indicated on the graphs (* *p* < 0.05). Circles: PRCP activity values for lean individuals; Squares: PRCP activity values for Obese individuals (**A**) or for Obese, non-diabetic individuals (**B**); Triangles: PRCP activity values for Obese, diabetic individuals; Boxes: Boxplots with 10-90 percentile.

**Figure 5:**
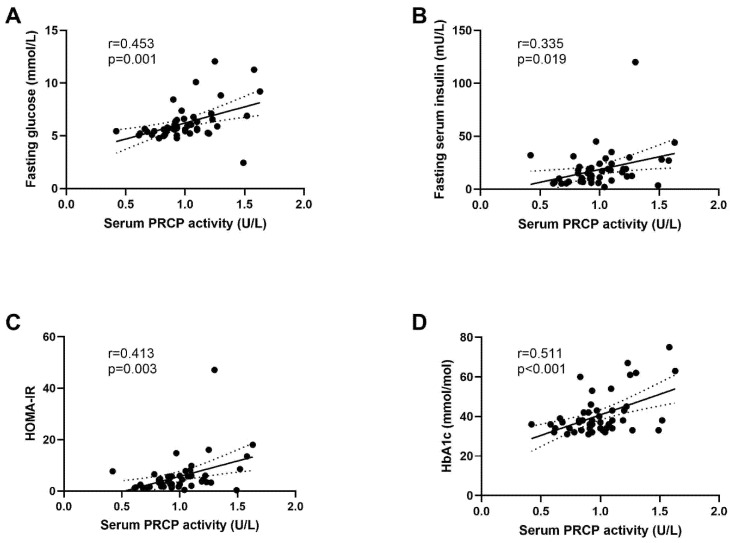
Correlation between serum PRCP activity and fasting glucose (**A**), fasting serum insulin (**B**), HOMA-IR (**C**) and HbA1c (**D**). Linear regression line is shown with 95% confidence interval. Circles: individual values; solid lines: Linear regression line; dotted line: 95% confidence interval for the regression line.

Lean and obese men showed a comparable serum PRCP activity (Figure 4A). Interestingly, when dividing obese men based on the presence or absence of T2D, mainly obese men with T2D showed significantly higher serum PRCP activity compared to their non-diabetic counterparts (*p* = 0.047) as well as to the lean individuals (*p* = 0.026) (Figure 4B). The association between serum PRCP activity and glycemic control was further confirmed, as significant positive correlations (Figure 5) were observed between serum PRCP activity and fasting glucose (r = 0.453, *p* = 0.001), fasting serum insulin (r = 0.335, *p* = 0.019), HOMA-IR (r = 0.413, *p* = 0.003) and glycated hemoglobin A1 (HbA1c, r = 0.511, *p* < 0.001) in the overall group. No significant correlations were found for variables related to body composition.

### 2.3. PRCP Activity Is Positively Correlated with Immune Cell Populations

**Figure 6:**
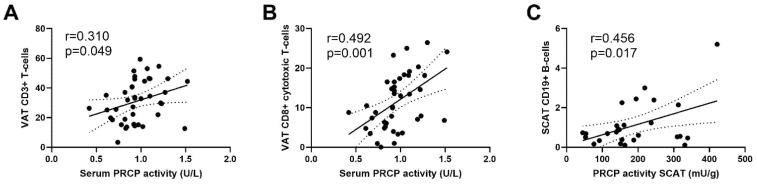
Correlation between serum PRCP activity and VAT CD3+ T-cells (**A**) and VAT CD8+ cytotoxic T-cells (**B**) and the correlation between PRCP activity in SCAT and SCAT CD19+ B-cells (**C**). Linear regression line is shown with 95% confidence interval. Circles: individual values; solid lines: Linear regression line; dotted line: 95% confidence interval for the regression line.

Serum PRCP activity was positively correlated with VAT CD3+ T-cells (r = 0.310, *p* = 0.049) and VAT CD8+ cytotoxic T-cells (r = 0.492; *p* = 0.001) but not with SCAT immune cell populations (Figure 6A,B and Supplementary Table S1). No correlations were found between serum PRCP activity and adipocyte size (r = 0.196 and *p* = 0.212 for SCAT and r = 0.091 and *p* = 0.547 for VAT).

PRCP activity in SCAT was positively correlated with SCAT CD19+ B-cells (r = 0.456, *p* = 0.017), while no significant correlations were found between VAT immune cell populations and VAT PRCP activity (Figure 6C and Supplementary Table S2). No correlations were found between PRCP activity in SCAT or VAT and adipocyte size (r = −0.029 and *p* = 0.875 for SCAT and r = −0.037 and *p* = 0.799 for VAT).

### 2.4. PRCP Activity Could Not Be Detected in Conditioned Media of Human Adipocytes

To investigate whether adipocytes are capable of secreting PRCP, we performed PRCP activity measurements in conditioned hMADS. PRCP activity in conditioned media of differentiated hMADS was below the limit of quantification (LOQ = 0.19 U/L), due to high dilution of secreted PRCP in these media or due to the fact that the PRCP activity measured in human adipose tissue does not derive from the adipocyte component but rather from the SVF.

## 3. Discussion

The present study shows for the first time that PRCP activity is present in human adipose tissue. PRCP activity is the highest in visceral adipose tissue, as compared to subcutaneous adipose tissue, and is only minimally present in skeletal muscle tissue in the overall group (lean and obese men with or without T2D). Serum PRCP activity levels are significantly elevated in obese men with T2D, as compared to lean and obese men without T2D. Furthermore, serum PRCP activity was positively correlated with different biochemical indicators of glycemic control (fasting glucose, serum insulin, HOMA-IR and HbA1c) and immune cell populations (CD3+ and CD8+ T-cells) in VAT.

Our data support the findings of Tabrizian et al., which showed that plasma PRCP concentrations are elevated in patients with uncontrolled diabetes and are normal in patients with controlled diabetes [16]. Interestingly, some differences were observed compared with our previous study about serum PRCP activity in obese and lean women [15]. In the present study, no significant difference was found in serum PRCP activity between lean and obese male subjects and no correlations were found between serum PRCP activity and metabolic parameters. In our previous study, in female subjects, an increasing serum PRCP activity was seen with an increasing BMI and serum PRCP activity was positively correlated with different metabolic parameters. Furthermore, in female subjects, no correlations were observed between serum PRCP activity and HbA1c and oral glucose tolerance test results [15]. This indicates a difference in the role of PRCP in glucose metabolism between male and female individuals, corroborating findings in animal studies, which showed differences in glucose metabolism between female and male PRCP-knockdown mice [10]. Interestingly, sexual dimorphism in human adipose distribution and adipose tissue function contributes considerably to sex differences in tissue-specific insulin sensitivity and cardiometabolic health [21], in which PRCP activity might thus play a role. A limitation of our present study is that we only included male individuals. Measurement of PRCP activity in adipose tissue of women will be interesting for comparison. PRCP protein concentrations were also reported to be higher in patients with obesity or diabetes in comparison with healthy controls, but in patients with both obesity and diabetes, the PRCP plasma concentrations were significantly higher than in patients with obesity only [14]. The latter is in line with the elevated serum PRCP activity seen in men with T2D in the current study.

To the best of our knowledge, this is the first time PRCP activity was measured in human adipose tissue. The observation that PRCP activity is significantly higher in VAT and SCAT in comparison with the PRCP activity in muscle tissue can indicate that circulating PRCP is originating from adipose tissue. However, the PRCP activity in adipose tissue was not correlated with serum PRCP activity. The higher PRCP plasma protein and serum PRCP activity levels seen in obese individuals in previous studies [14,15] can be explained by the fact that obese individuals have more SCAT and VAT mass as compared to lean individuals. To investigate whether adipocytes are capable of secreting PRCP, we measured PRCP activity in conditioned media derived from differentiated hMADS. However, PRCP was undetectable with our in-house validated assay. Indicating PRCP might be too much diluted in the conditioned media or PRCP activity measured in adipose tissue does not derive from adipocyte component, but rather from the SVF, including immune cells, of adipose tissue. The latter is strengthened by the positive correlations between serum PRCP activity and immune cell populations in the SVF, like T-cells. Moreover, PRCP activity has been observed in these cell populations [15] and elevated serum/plasma PRCP is already described in several diseases with an inflammatory component such as rheumatoid arthritis, stroke and sepsis [17,18,19]. Furthermore, an upregulation of PRCP mRNA expression was seen in human lipopolysaccharide-stimulated endothelial cells [20] and pro-inflammatory stimulation of human endothelial cells also induced PRCP secretion, while its intracellular activity level remained constant [3]. Interestingly, serum PRCP activity was positively correlated with VAT immune cell populations and not with SCAT immune cell populations. The latter might be explained by the different immune environments in SCAT and VAT. VAT is known to be more inflammatory and shows more immune cell infiltration compared to SCAT. Further research is warranted to find out which adipose tissue resident cells are capable of secreting PRCP, in order to gain further insight in the origin of circulating PRCP.

## 4. Materials and Methods

### 4.1. Study Design

Lean and obese age-matched men of Caucasian origin who were scheduled to undergo laparoscopic abdominal (inguinal hernia or gallbladder removal) or bariatric surgery were recruited, as described previously [22]. The lean control group involved 19 male subjects. The obese group (BMI > 30 kg/m2) consisted of 36 male subjects, including 25 individuals without T2D and 11 individuals with T2D. Presence of T2D was based on known clinical diagnosis (on average 2.5 years of diagnosis, ranging from newly diagnosed to 6 years). Diabetic men had glycated hemoglobin A1 (HbA1c) levels ≥ 6.5% (45 mmol/mol) or were on glucose lowering medication. From these subjects, VAT, SCAT and muscle tissue and serum samples were collected (see below). The study protocol was approved by the Medical Ethical Committee Jessa hospital, Hasselt, and Hasselt University, Belgium, in agreement with the Declaration of Helsinki (2008). Before participating in the study, all individuals gave their written informed consent. 

### 4.2. Anthropometric Measurements and Blood Sampling

Body weight, height, waist/hip circumference and blood pressure were measured at the morning of surgery. Venous blood samples were collected after an overnight fast. Different metabolic and biochemical parameters were determined as described previously [22,23]. Characteristics of the different subjects are shown in Table 1.

### 4.3. Abdominal Subcutaneous and Visceral Adipose Tissue Biopsies

After an overnight fast, SCAT and VAT biopsies were collected from the periumbilical subcutaneous adipose tissue depot and the distal portion of the omentum majus, respectively. Muscle tissue was collected from the musculus rectus abdominis. The tissue samples were immediately placed in saline and transported on ice to the laboratory for further processing. A portion of the fresh adipose tissue was frozen in liquid nitrogen and kept at −80 °C until further processing for PRCP activity measurements. Another portion of the adipose tissue was used for isolation of the SVF and flow cytometry analyses. 

### 4.4. Tissue Lysate Preparation

Tissue biopsies were ground to a fine powder under liquid nitrogen and kept at −80 °C until further processing. On the day of activity measurement or Western blot analysis, the powder was dissolved in 2 µL of lysis buffer per mg tissue for activity measurement (1% octyl glucoside, 10 mM EDTA, 70 µg/mL aprotinin, 50 mM Tris, pH 8.3) or for Western blot (1% Triton X-100, 150 mM NaCl, Protease inhibitor cocktail (Roche, Basel, Switzerland), 50 mM Tris, pH 7.6). Samples were kept on ice for 1 h with frequent agitation. Subsequently, samples were centrifuged for 30 min at 12,000× *g* at 4 °C. For adipose tissue, the infranatant, the transparent layer between the upper fat layer and the lower cell debris, was collected and immediately used for activity measurement or Western blot analysis. For skeletal muscle tissue, supernatant was collected and immediately used for activity measurement or Western blot analysis. Protein concentrations were determined via the Bradford method with bovine serum albumin as the standard protein.

### 4.5. PRCP Activity Measurement

PRCP activity was measured using a validated reverse phase high performance liquid chromatography (RP-HPLC) method [24]. In brief, 10 µL of each sample (serum, tissue lysate or conditioned media) was incubated in duplicate with 75 µL 8 mM Z-Pro-Phe at pH 5 (0.1 M Natrium Acetate, 10 mM EDTA) for 2 h at 37 °C. To stop the enzymatic reaction, 75 µL stop solution (10% perchloric acid and 20% acetonitrile in purified water) was added and samples were centrifuged at 12,000× *g* for 10 min at 4 °C. Ten µL of the supernatant was injected into the RP-HPLC system (Shimadzu) and the enzymatically formed Z-Pro was determined by its UV absorbance at 210 nm. Quantification was performed by peak height measurements. The PRCP activity is expressed as units per gram (U/g) protein for the tissue lysate or units per liter (U/L) for serum. One unit defines the amount of enzyme that hydrolyses 1 μmol of substrate per minute. 

### 4.6. Western Blot

Tissue lysates were diluted in Laemmli sample buffer (4×; 40% glycerol, 240 mM Tris-HCl pH 6.8, 8% SDS, 0.04% bromophenol, 5% β-mercaptoethanol) and boiled for 5 min before loading onto a 10% SDS-PAGE gel. 20 µg of protein was loaded for each tissue lysate. Electrophoresis was performed at 180 V for 60 min in the Mini-Protean III system (Bio-Rad). Next, the proteins were transferred to nitrocellulose membranes by electroblotting in 25 mM Tris buffer pH 8.3 with 0.192 M glycine and 20% methanol in the Mini Trans-blot Cell assembly (Bio-Rad, Hercules, CA, USA) for 60 min at 250 mA. Subsequently, the membranes were blocked in 5% skimmed milk in washing buffer (0.05 M Tris, 0.15 M NaCl, 0.01% Tween 20, pH 7.4) for 1 h at room temperature. The membranes were incubated overnight at 4 °C with primary antibodies diluted in blocking buffer: anti-human PRCP (HPA 017065, Sigma-Aldrich, St. Louis, MO, USA, 1:500) and anti-vinculin as loading control (ab129002, Abcam, 1:20,000). Next, the membranes were incubated with secondary antibody (Goat-anti-Rabbit HRP, Invitrogen, Waltham, MA, USA, 1:5000 in blocking buffer) for 2 h at room temperature. Between the different incubations, membranes were washed 5 × 5 min with washing buffer. Chemiluminescent detection was performed using the SuperSignal West Femto substrate kit (ThermoFisher Scientific, Waltham, MA, USA) and the membrane was visualized via an OptiGo viewer and Proxima AQ-4 Software version 1.29. Precision Plus Protein Dual Color Standards (Bio-Rad) were used for molecular weight estimation. WesternSure pen (LI-COR, Lincoln, NE, USA) was used to visualize the protein ladder. Quantification was performed in relation to vinculin via ImageJ.

### 4.7. Flow Cytometry

Multicolor flow cytometry measurements were performed and analyzed as described previously [23,25]. Briefly, cells isolated from the SVF, which were obtained following collagenase digestion and lysis of red blood cells, were stained for flow cytometry using two antibody cocktails. Cocktail 1 included CD45-PE-Cy7 (BD 557748), CD3-fitc (BD 561807), CD19-fitc (BD 555412), CD56-fitc (BD 562794), CD66b-fitc (BD 555724), CD11b-BV421 (Biologend 301324), and CD11c-APC-Cy7 (Biolegend 337218). Cocktail 2 included CD45-PE-Cy7 (BD 557748), CD3-V500 (BD 561416), CD4-PerCP (Biolegend 300528), CD8 APC-H7 (BD 641400), CD19-BV421 (Biolegend 302234), and CD56-APC (Biolegend 318310). Samples were measured with a FACS-Canto II (BD Biosciences, Gurugram, India). Results were analyzed with FACSdiva (BD Biosciences) and FlowJo software version 10. Since weight of the adipose tissues was unavailable, data are expressed as % of cells relative to total cells (based on forward and side scatter plot). 

### 4.8. Adipocyte Size

Adipocyte size measurement was performed as described previously [23,26]. A part of the adipose tissue samples was fixed overnight in 4% paraformaldehyde and embedded in paraffin. Histological sections (8 µm) were cut, mounted on microscope glass slides and dried overnight in an incubator at 37 °C. Hematoxylin and eosin staining was used. Digital images were captured using a Leica DFC320 digital camera (Leica DM3000 microscope) at 20× magnification. Adipocyte size and distribution was assessed in a blinded fashion (coefficient of variation < 5%) using computerized morphometric analysis (Leica QWin V3) of individual adipocytes (at least 400 adipocytes per sample).

### 4.9. Conditioned Media of Human Adipocytes

Human multipotent adipose-derived stem cells (hMADS), a validated human white adipocyte model, were obtained from adult lean (*n* = 10) and obese (*n* = 10) donors (different from the groups above) and differentiated into the adipogenic lineage. Cells were seeded at a density of 2000 cells/cm^2^ and cultured as described before [27]. At day 11 to 14 of differentiation, supernatant was collected, centrifuged for 5 min at 300× *g* at 4 °C and aliquoted in 600 µL fractions, snap-frozen in liquid nitrogen and stored at −80 °C until further analysis.

### 4.10. Statistical Analysis

Data were analyzed using the statistical SPSS software version 27 (IBM). Two-way ANOVA analysis was conducted to examine the effect of tissue type (VAT, SCAT and skeletal muscle tissue) and group (obese, with and without T2D, and lean individuals) on PRCP activity. Tukey Post hoc analysis was conducted for variables that showed significant main effects. A Mann–Whitney U test was performed to analyze the difference in PRCP protein expression between lean and obese individuals. A student *t*-test for unpaired samples was used to compare PRCP activity in serum of obese and lean individuals. A one-way ANOVA, followed by a post hoc analysis using Bonferonni correction for multiple comparisons, was carried out to assess differences in serum PRCP activity between groups. The relation between PRCP activity and several biochemical and metabolic parameters was described using Pearson’s correlation coefficient. Potential correlations between PRCP activity, adipocyte size and frequency of immune cell populations in the SVF of SCAT and VAT were assessed using Pearson’s correlation coefficient. PRCP activity levels in serum are presented as boxplots (10–90 percentile) and PRCP activity levels in tissue are presented as mean ± SEM (GraphPad Prism 9 software). Statistical significance was set at *p* < 0.05. 

## 5. Conclusions

In conclusion, PRCP activity was demonstrated for the first time in human adipose tissue and serum PRCP activity is associated with glycemic control in obese men. In both lean and obese individuals, PRCP activity is significantly higher in SCAT and VAT as compared to the activity in muscle tissue. Moreover, a positive correlation was seen between serum PRCP activity and VAT immune cell populations and between SCAT PRCP activity and SCAT CD19+ B-cells. These results may indicate that circulating PRCP activity is resulting from the adipose tissue immune cell fraction rather than from adipocytes. The role of PRCP in adipocytes and PRCP’s exact origin deserves further investigation, especially because adipocytes produce and secrete the PRCP-substrate (pyr)-apelin-13.

## Figures and Tables

**Table 1:** Characteristics of lean, obese non-diabetic and obese diabetic men. Data are expressed as mean ± SD.

Variable	Lean Individuals	Obese, Non-Diabetic Individuals	Obese, Diabetic Individuals
*n*	19	25	11
Age (years)	52 ± 6	50 ± 7	52 ± 7
Length (m)	1.80 ± 0.07	1.80 ± 0.07	1.76 ± 0.04
Weight (kg)	78 ± 7	124 ± 15	117 ± 9
BMI (kg/m^2^)	24 ± 1	38 ± 4	38 ± 4
Waist circumference (cm)	91 ± 5	129 ± 10	126 ± 7
Hip circumference (cm)	94 ± 4	117 ± 9	116 ± 7
Waist-to-hip ratio	0.98 ± 0.03	1.09 ± 0.04	1.09 ± 0.06
Systolic blood pressure (mmHg)	125 ± 15	143 ± 14	147 ± 15
Diastolic blood pressure (mmHg)	80 ± 9	84 ± 8	85 ± 10
Fasting glucose (mmol/L)	5.7 ± 0.8	6 ± 1	8 ± 2
Serum insulin (mU/L)	7 ± 4	21 ± 10	30 ± 31
HOMA-IR	2 ± 1	6 ± 3	12 ± 13
HbA1c (%)	5.2 ± 0.2	5.8 ± 0.7	7.0 ± 1.0
HbA1c (mmol/mol)	33 ± 3	40 ± 8	56 ± 11
Fat percentage (%)	23 ± 4	37 ± 5	37 ± 3
Fat mass (kg)	18 ± 4	46 ± 11	43 ± 7
Fat free mass (kg)	58 ± 6	76 ± 7	74 ± 4
NEFA (µmol/L)	244 ± 169	759 ± 319	872 ± 301
Triacylglycerols (mmol/L)	907 ± 587	1274 ± 407	1271 ± 524

BMI: body mass index; HOMA-IR: Homeostatic Model Assessment for Insulin Resistance; HbA1c: glycated hemoglobin A1; NEFA: non-esterified fatty acids.

## Data Availability

The datasets generated during and/or analyzed during the current study are available from the corresponding author on reasonable request.

## Supplementary Materials

The supporting information can be downloaded at: https://www.mdpi.com/article/10.3390/ijms232113529/s1.
