# Supplementary material for: Prolyl Carboxypeptidase Activity Is Present in Human Adipose Tissue and Is Elevated in Serum of Obese Men with Type 2 Diabetes
Source: Int J Mol Sci. 2022 Nov 4;23(21):13529. doi: 10.3390/ijms232113529 (PMC9655216; doi:10.3390/ijms232113529)
Supplement: Supplementary file 1 [file ijms-23-13529-s001.zip › ijms-1991251-supplementary.pdf]

Supplementary Table S1: Pearson correlations between serum PRCP activity and SCAT and VAT immune cell populations.

	r SCAT	p SCAT	r VAT	p VAT
Total CD45+ leukocytes, % of total cells	-0.200	0.215	0.096	0.550
CD11c+ M1 macrophages, % of total cells	-0.158	0.332	-0.155	0.334
CD11c- M2 macrophages, % of total cells	-0.064	0.694	-0.056	0.726
M1/M2 ratio	-0.184	0.255	0.192	0.229
CD56+ NK cells, % of total cells	-0.028	0.866	0.139	0.386
CD3+ T-cells, % of total cells	0.063	0.700	**0.310**	**0.049**
CD3+ CD4+ T-helper cells, % of total cells	0.038	0.816	0.006	0.971
CD3+ CD8+ cytotoxic T-cells, % of total cells	0.154	0.342	**0.492**	**0.001**
CD19+ B-cells, % of total cells	-0.042	0.799	0.039	0.809

Supplementary Table S2: Pearson correlations between SCAT PRCP activity and SCAT immune cell populations and Pearson correlations between VAT PRCP activity and VAT immune cell populations.

	r SCAT	p SCAT	r VAT	p VAT
Total CD45+ leukocytes, % of total cells	-0.256	0.197	0.140	0.370
CD11c+ M1 macrophages, % of total cells	-0.112	0.577	-0.008	0.957
CD11c- M2 macrophages, % of total cells	-0.116	0.564	-0.157	0.314
M1/M2 ratio	0.036	0.859	0.140	0.372
CD56+ NK cells, % of total cells	0.130	0.519	0.022	0.891
CD3+ T-cells, % of total cells	0.024	0.907	0.179	0.252
CD3+ CD4+ T-helper cells, % of total cells	0.016	0.935	0.110	0.482
CD3+ CD8+ cytotoxic T-cells, % of total cells	-0.126	0.533	0.171	0.273
CD19+ B-cells, % of total cells	**0.456**	**0.017**	0.181	0.245
